# The Effect of Preconditioning Strategies on the Adsorption of Model Proteins onto Screen-Printed Carbon Electrodes

**DOI:** 10.3390/s22114186

**Published:** 2022-05-31

**Authors:** Tea Romih, Ivan Konjević, Lea Žibret, Ika Fazarinc, Ajda Beltram, David Majer, Matjaž Finšgar, Samo B. Hočevar

**Affiliations:** 1 Department of Analytical Chemistry, National Institute of Chemistry, Hajdrihova 19, SI-1000 Ljubljana, Slovenia; tea.romih@ctk.uni-lj.si (T.R.); lea.zibret@zag.si (L.Ž.); if2912@student.uni-lj.si (I.F.); ab9644@student.uni-lj.si (A.B.); 2 Faculty of Chemistry and Chemical Technology, University of Maribor, Smetanova 17, SI-2000 Maribor, Slovenia; ivan.konjevic@student.um.si (I.K.); david.majer@um.si (D.M.); matjaz.finsgar@um.si (M.F.)

**Keywords:** screen-printed carbon electrode, SPE, electrochemical biosensor, adsorption, bovine serum albumin, protein A, glow discharge

## Abstract

The preconditioning and modification of the supporting electrode surface is an essential step in every biosensor architecture. In particular, when using screen-printed carbon electrodes (SPEs) as inexpensive and convenient disposable sensor substrates, their somewhat lower electrochemical (surface) reproducibility might represent a complex hurdle. Herein, we investigated the effect of selected preconditioning strategies, such as cyclic voltammetric pretreatment, in H_2_SO_4_ and H_2_O_2_ and plasma pretreatment with a positive and negative glow discharge, which all improved the electrochemical stability of the unmodified SPEs. Furthermore, we studied the influence of preconditioning strategies on the adsorption kinetics of the two most commonly used building blocks for biosensor preparation, i.e., bovine serum albumin (BSA) and protein A. We observed an advantageous effect of all the examined preconditioning strategies for the modification of SPEs with protein A, being the most effective the negative glow discharge. On the other hand, BSA exhibited a more complex adsorption behavior, with the negative glow discharge as the only generally beneficial preconditioning strategy providing the highest electrochemical stability. Protein A revealed a more substantial impact on the electrochemical signal attenuation than BSA considering their same concentrations in the modification solutions. For both BSA and protein A, we showed that the concentrations of 5 and 10 μg mL^−1^ already suffice for an electrochemically satisfactorily stable electrode surface after 60 min of incubation time, except for BSA at the positive-plasma-treated electrode.

## 1. Introduction

Adsorption is one of the simplest strategies for building the sensing layers of electrochemical biosensors. Usually performed by drop-casting, adsorption-based techniques are fast, allow low reagent consumption, and require only basic training of the experimenter. They can be performed under mild reaction conditions and are minimally disruptive to the biomolecules. Besides chemisorption, a large number of these techniques rely on physisorption to hold biomolecules in place through Van der Waals interactions, which are highly dependent on pH, temperature, and solvent properties, and may be reversible. On the other hand, other construction methodologies (e.g., entrapment in a polymer, crosslinking by a multifunctional reagent, covalent binding) have some specific disadvantages, particularly complex and time-consuming procedures, and a high risk of biomolecule damage [1]. Adsorption-based techniques for the construction of biosensors may thus still be preferable in certain cases.

When the biomolecules in question are proteins, their adsorption on solid surfaces is governed by an interplay of the surface, protein, and solution characteristics. Protein adsorption on the electrode surface, regarding both its amount and orientation, affects the biosensor’s sensitivity, specificity, and stability and thus needs to be properly controlled [2]. Besides optimizing protein concentration and solution components, an important factor to consider is the preconditioning of the electrode surface, particularly when using screen-printed (carbon) electrodes (SPEs). In contrast to solid-state working electrodes, SPEs usually cannot be polished, so the desired characteristics of the electrode surface need to be achieved in other ways. Potential cycling in oxidative solutions, e.g., in sulfuric acid (H_2_SO_4_) or hydrogen peroxide (H_2_O_2_), is a commonly employed method to remove organic crosslinks from the carbon ink, increasing surface roughness and functionality and, in turn, improving the electrode performance along with its reproducibility [3]. Similar results can be achieved with oxygen plasma treatment [4], an option that has become increasingly feasible with the advent of compact, benchtop plasma treatment instruments in recent years.

In building electrochemical biosensors, certain proteins stand out regarding frequency of use and their particular role in the sensing layer architecture. Two such proteins are bovine serum albumin (BSA) and protein A. BSA is often used as one of the building blocks for complex biocomposite modification layers [5] or as a filler biomolecule used to block exposed binding sites during modification to prevent non-specific binding [6]. BSA belongs to the class of serum albumins, the most abundant proteins in mammal blood, which are responsible for maintaining colloid osmotic blood pressure and blood pH. Because of their abundance, low cost, and inherent stability, serum albumins are one of the most extensively studied and applied protein classes in biochemistry and often serve as “model” proteins [7]. BSA contains 583 amino acids, which amount to a total molecular weight of approximately 66 kDa. Its isoelectric point is between pH 4.5 and 4.8 [8].

On the other hand, protein A is a component of the cell wall of *Staphylococcus aureus* and binds several mammalian immunoglobulins by their Fc region (antigen-binding site). Its primary role in *S. aureus* is to bind the host’s IgG antibodies in such a way that they are oriented away from the bacterium, which hinders the attack by the host’s immune system. In biochemistry, this feature is exploited for binding antibodies (usually IgG) in an oriented manner, for example, in immunoassays or affinity chromatography [9]. It can also be used as a versatile building block in the development of electrochemical immunosensors [10]. Protein A’s molecular weight is 42 kDa, and its isoelectric point is around pH 5.1 [11].

The impetus of this study was to investigate the effects of different preconditioning strategies on the electrochemical stability of commercial SPEs and particularly on the degree and kinetics of adsorption of BSA and protein A onto the electrode surface. Herein, we discuss the advantages and disadvantages of each approach and the optimal modification conditions for the purpose of simple, adsorption-based construction of electrochemical biosensors using BSA or protein A as a base layer.

## 2. Materials and Methods

### 2.1. Materials and Reagents

Sulfuric acid (96%), H_2_O_2_ (30%), NaCl, KCl, and KH_2_PO_4_ were purchased from Merck (Darmstadt, Germany), IgG-free BSA powder, K_3_[Fe(CN)_6_] and K_4_[Fe(CN)_6_] from Sigma Aldrich (St. Louis, MO, USA), and Na_2_HPO_4_∙2H_2_O from VWR Chemicals (Radnor, PA, USA), and were of the analytical grade. Native *Staphylococcus aureus* protein A (ab7399) solution was purchased from Abcam (Cambridge, UK) and stored in a freezer at −20 °C until further use. All solutions used in this work were prepared using ultrapure water, with a resistivity of 18.2 MΩ cm, obtained from the Milli-Q system (Millipore, Darmstadt, Germany). K_3_[Fe(CN)_6_] and K_4_[Fe(CN)_6_] were diluted to 1 mM + 1 mM mixture in 0.1 M KCl. Phosphate-buffered saline (PBS) was prepared according to the recipe from AAT Bioquest [12]. Protein A and BSA solutions were prepared in PBS without preservatives and stored in low-protein-binding test tubes in a freezer at −20 °C for longer periods or in a refrigerator at 2 °C during experiments.

### 2.2. Electrochemical Apparatus

Voltammetric experiments were carried out in a 10 mL glass electrochemical cell using a portable PalmSens4 potentiostat/galvanostat (PalmSens BV, Houten, The Netherlands) in combination with a cable connector for SPEs (DRP-CAC 71606, Metrohm DropSens, Asturias, Spain). The instrument was controlled with the PSTrace 5.8 software (PalmSens BV). All measurements were performed at laboratory temperature (22–23 °C).

Electrochemical impedance spectroscopy (EIS) measurements have been performed using Autolab PGSTAT204 potentiostat/galvanostat (Metrohm Autolab, Utrecht, The Netherlands). Measurements were performed in a frequency range from 100 kHz to 20 mHz with 8 points per decade and a 10 mV (peak-to-peak) amplitude of the excitation signal. EIS measurements were performed in a solution containing 10 mM K_3_[Fe(CN)_6_] (99.9%, Sigma Aldrich, St. Louis, MO, USA) and 0.1 M KCl (p.a., Carlo Erba Reagents, Milan, Italy).

Commercial SPEs type DRP-C110 were purchased from Metrohm DropSens. These electrodes have a single circular carbon working electrode (4 mm in diameter), a carbon counter electrode, and a silver quasi-reference electrode and are suitable for use in a solution.

### 2.3. Preconditioning of SPEs

Several electrode preconditioning strategies were tested to discern the one that would yield the most reproducible protein adsorption on the supporting working electrode. Each electrode was subjected to only one preconditioning strategy. The effects of preconditioning were compared to the characteristics of new (pristine) electrodes.

#### 2.3.1. Potential Cycling in H_2_SO_4_

The protocol for electrode preconditioning with H_2_SO_4_ was adapted from Pine Research [3]. The H_2_SO_4_ concentration was optimized to achieve the highest measurement reproducibility at a single electrode. The following parameters were finally chosen for the electrode preconditioning: 1 mM H_2_SO_4_, three voltammetric cycles in a potential range of −1.5 V to 1.5 V, a step potential of 0.01 V, and a scan rate of 0.1 V/s. Hereafter, we refer to these electrodes as H_2_SO_4_-treated electrodes.

#### 2.3.2. Potential Cycling in H_2_O_2_

The protocol for electrode preconditioning with H_2_O_2_ was adapted from González-Sánchez et al. [13] and performed as follows: 10 mM H_2_O_2_ in PBS with pH 7.0, 25 voltammetric cycles in a potential range of −0.7 V to 1.0 V, a step potential of 0.01 V, and a scan rate of 0.1 V/s. Hereafter, we refer to these electrodes as H_2_O_2_-treated electrodes.

#### 2.3.3. Positive and Negative Glow Discharge

The electrodes were subjected to either positive or negative glow discharge produced by the GloQube^®^ Plus Glow Discharge System (Quorum Technologies, Lewes, UK), a compact and fully automated system for transmission electron microscope (TEM) grids and surface modification. The electrodes were laid with the sensing side up into a glass Petri dish and placed into the in-air modification chamber before applying glow discharge. Apart from the positive or negative charge, the plasma current (1 mA), plasma process time (30 s), and loading tray height (medium) were the same for both modification procedures. In continuation, we refer to these electrodes as positive-plasma-treated and negative-plasma-treated electrodes.

### 2.4. Assessment of Electrochemically Active Surface Area

Three electrodes per each preconditioning strategy were used to estimate the electrochemically active surface area using the peak heights of cyclic voltammograms vs. square root of the scan rate (*υ*^½^) [14]. The electrochemical probe used was the same as in other experiments, i.e., 1 mM + 1 mM mixture of K_3_[Fe(CN)_6_] and K_4_[Fe(CN)_6_] in 0.1 M KCl. The diffusion coefficients of K_3_[Fe(CN)_6_] and K_4_[Fe(CN)_6_] in 0.1 M KCl were taken from Konopka and McDuffie [15].

### 2.5. Protein Adsorption Electrochemical Experiments

BSA and protein A solutions were diluted with PBS to 5 μg mL^−1^, 10 μg mL^−1^, and 20 μg mL^−1^ in low-protein-binding test tubes. Each working electrode was covered with 10 μL of either solution and incubated in a humid atmosphere for 5, 15, 30, 45, or 60 min. Three electrodes were sampled per each combination of variables (preconditioning strategy, protein type, its concentration, and incubation time), and each electrode was incubated only once. A single square-wave voltammetric (SWV) scan in a potential range of −0.2 V to 0.7 V was recorded in 10 mL of a 1 mM + 1 mM mixture of K_3_[Fe(CN)_6_] and K_4_[Fe(CN)_6_] in 0.1 M KCl, before the incubation and again after the incubation. The signal attenuation due to protein adsorption was expressed as *I*/*I*_0_, where *I*_0_ is the voltammetric peak height at a bare electrode, and *I* is the voltammetric peak height at the same electrode after incubation with a protein.

### 2.6. Assessment of the Mass of Adsorbed Proteins with FluoroRed 600 Fluorescent Dye

A separate group of electrodes was used to assess the influence of electrode preconditioning on the mass of adsorbed proteins with the FluoroRed 600 fluorescence-based protein quantification assay (G-Biosciences No. 786-1596). The electrodes were incubated with either 10 µL of BSA or protein A at a concentration of 10 μg mL^−1^ or 20 μg mL^−1^ for 60 min in the same way as for the protein adsorption electrochemical experiments (Section 2.5). Two electrodes were sampled per each combination of variables. After the incubation, 40 µL of the 1× reaction buffer (diluted with deionized water from the 10× FluoroRed 600 Reaction Buffer included in the kit) was added onto the working electrode of each SPE. The liquid was mixed through a few repeated aspirations and expulsions from the pipette tip, and then the entire 50 µL of the mixture was aspirated into the pipette tip and transferred into a black 96-well microtiter plate (Greiner Bio-One No. 675076). Additional 50 µL of the 1× reaction buffer were added to each well, followed by 100 µL of the FluoroRed 600 Dye Reaction Reagent according to the manufacturer’s instructions. A calibration curve was prepared in duplicate for both proteins, i.e., the BSA standard included in the kit and the Abcam’s protein A stock according to the manufacturer’s instructions. The plate was shaken while encased in a foam stand for microtiter plates on a VELP Scientifica CLASSIC vortex mixer (Usmate Velate, Italy) in the dark for 30 min at laboratory temperature.

The samples’ absorbances were read at 490 nm and emissions at 600 nm with a Synergy Mx multi-mode microplate reader (BioTek, Winooski, VT, USA). The protein concentrations in the droplets aspirated from the electrodes were calculated from the calibration curve, and the mass of adsorbed proteins was calculated as the difference between the mass of added proteins before incubation and the mass of aspired proteins after incubation.

### 2.7. X-ray Photoelectron Spectroscopy (XPS)

High-resolution XPS measurements were performed with the AXIS Supra+ device (Kratos, Manchester, UK) at a pass energy of 20 eV. The size of the analysis spot was 700 μm by 300 μm. Al K_α_ spectral line served as the excitation source, and the C–C/C–H peak in the C 1s at 284.8 eV was used to correct the binding energy (*E*_B_) scale. Data were acquired and processed using the ESCApe program (Kratos, Manchester, UK). Measurements were performed at 90° take-off angle. Shirley background subtraction was employed, and surface composition was determined as atomic concentrations normalized to 100.0%.

## 3. Results and Discussion

### 3.1. The Effect of Surface Preconditioning on the Physico-Chemical Characteristics of the SPE Surface

The electrochemically active surface areas of pristine and preconditioned electrodes were assessed from cyclic voltammograms of K_3_[Fe(CN)_6_]/K_4_[Fe(CN)_6_] (Figure S1) based on Randles–Ševčík equation:

*I*_p_ = k n^3/2^
*A* D^1/2^
*c*^b^
*υ*^1/2^ (1)

where the constant k = 2.69 × 10^5^; n is the number of moles of electrons electrochemically transferred per mole of electroactive species; *A* is the area of the electrode in cm^2^; D is the diffusion coefficient in cm^2^ s^−1^; *c*^b^ is the bulk solution concentration in mol L^−1^; and *υ* is the potential scan rate in V s^−1^.

The DRP-C110 SPEs from Metrohm Dropsens have working electrodes with a diameter of 4 mm; therefore, their geometric surface area (not accounting for surface roughness) is 12.57 mm^2^. The electroactive surfaces were determined to be 8.38 ± 0.57 mm^2^ for pristine electrodes, 6.45 ± 0.04 mm^2^ for H_2_O_2_-treated electrodes, 6.67 ± 0.58 mm^2^ for H_2_SO_4_-treated electrodes, 7.47 ± 0.07 mm^2^ for positive-plasma-treated electrodes, and 7.05 ± 0.53 mm^2^ for negative-plasma-treated electrodes (average ± SD, *n* = 3). The discrepancy between geometric and electroactive surface area is due to the conductive paste containing a dielectric binder that hinders the electron transfer across the working electrode. In addition, when the net surface charge becomes more negative after preconditioning, the electrode surface repels the negatively charged [Fe(CN)_6_]^3−^/[Fe(CN)_6_]^2−^ ions to a greater extent, and the electroactive surface area apparently becomes somewhat smaller. The effect of preconditioning on the electrode surface characteristics seems complex, so the observed changes in the electroactive surface area are likely caused by an interplay of different factors. Most importantly, the electrochemical response becomes stable after preconditioning together with significantly enhanced electrode-to-electrode reproducibility; moreover, the positive and negative discharge exhibited the most favorable effect on the stability and reproducibility of the SPE surface, whereas the preconditioning with H_2_SO_4_ and particularly with H_2_O_2_ exhibited somewhat lower effects; these results are summarized in Figures S2–S5.

**Figure 1:**
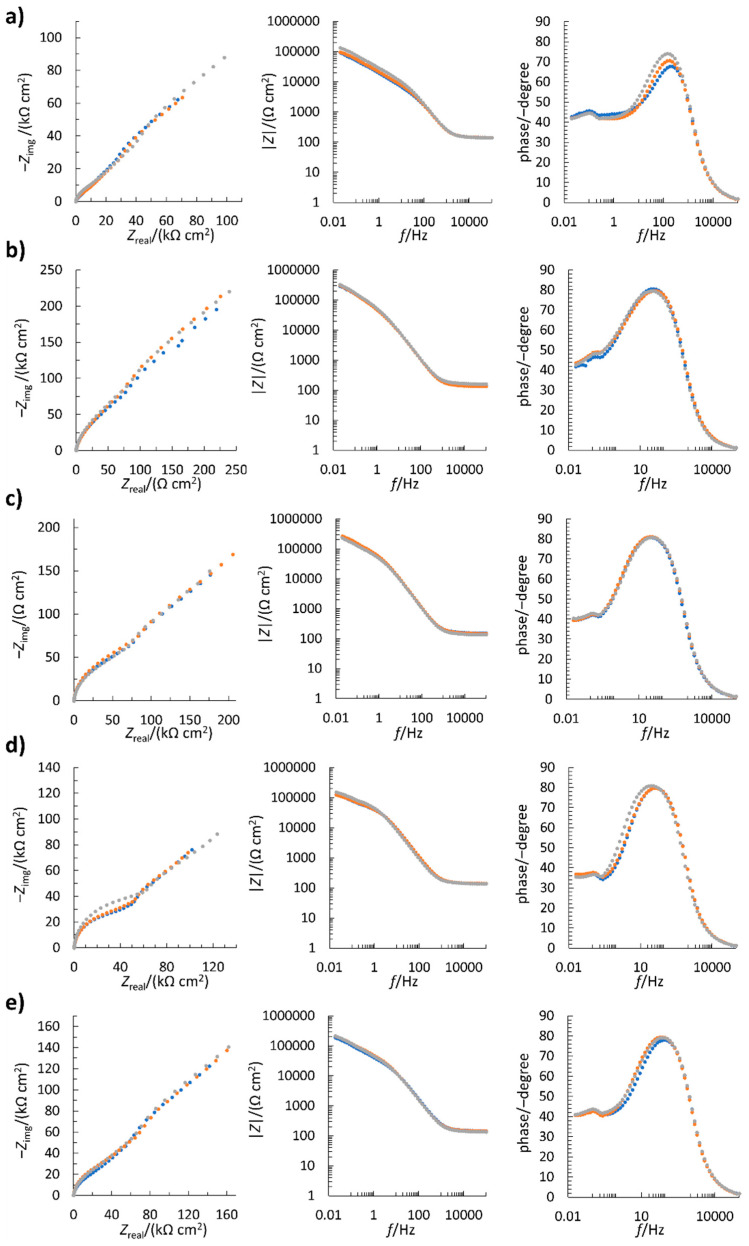
Electrochemical impedance spectroscopy measurements (three colors; three replicates) of differently treated electrodes in the solution of 10 mM K_3_[Fe(CN)_6_] and 0.1 M KCl. (**a**) Pristine electrode; (**b**) negative-plasma-treated electrode; (**c**) positive-plasma-treated electrode; (**d**) H_2_O_2_-treated electrode; (**e**) H_2_SO_4_-treated electrode.

Electrochemical impedance spectroscopy measurements are shown in Figure 1 (Nyquist spectra on the left, Bode modulus impedance spectra in the middle, and Bode phase spectra on the right). Three replicate measurements were performed for each electrode, resulting in a high degree of repeatability (Figure 1). The shape of the Bode modulus impedance spectra and Bode phase spectra in the middle-frequency range indicate the capacitive behavior of the double layer and the charge transfer resistance. In the low-frequency range, the deviation from the *x*-axis (Z_real_) in the Nyquist spectra and the phase angle approaching 45° in the Bode phase spectra indicate a diffusion-controlled process for each electrode. The equivalent electrical circuit of such a system can be characterized by R_s_(C_dl_(R_ct_W)), where R_s_, C_dl_, R_ct_, and W stand for solution resistance, double layer capacitance, charge transfer resistance, and Warburg diffusion, respectively. Based on this, it can be concluded that the electrochemical process is both kinetically and diffusion controlled for all five electrodes.

### 3.2. The Effect of Surface Preconditioning on the Amount of Adsorbed Proteins

The effect of the surface preconditioning strategy on protein adsorption efficiency and kinetics was assessed with a commonly employed depletion approach [2]. This technique requires a highly accurate and precise quantification procedure because the amount of adsorbed proteins per surface area is usually low. To this end, we employed one of the most sensitive protein assays currently available on the market, i.e., the FluoroRed 600 fluorescence-based protein quantification assay [16]. As per the supplier’s information, the linear concentration range of this assay is 0.05–5.00 μg mL^−1^, which is up to 500 times more sensitive than the classic bicinchoninic acid assay, the latter exhibiting a linear range of 25–2000 µg mL^−1^ [17]. The amount of adsorbed proteins was calculated by subtracting the mass of aspirated proteins determined with the assay from the mass of proteins added to the electrodes before incubation.

Table 1 shows that the percentages of adsorbed BSA at the lower applied concentration (i.e., 10 μg mL^−1^) were similar for all preconditioning strategies, except for the H_2_O_2_-treated electrodes, and ranged from 81% to 87%. For the H_2_O_2_-treated electrodes, the percentage of adsorbed BSA was slightly higher, namely 92%. These values were comparable with those for protein A (including the H_2_O_2_-treated electrodes with over 98% adsorption) at the same applied concentration, except for positive-plasma-treated electrodes, which adsorbed only 61% of the incubated protein A. However, at the higher applied concentration (i.e., 20 μg mL^−1^), the difference between BSA and protein A became more significant. While the percentages of adsorbed BSA remained in roughly the same range as at the lower applied concentration (i.e., resulting in ca. doubled masses of adsorbed protein at the SPE), the percentages of adsorbed protein A markedly decreased, reaching as low as 33% for the H_2_SO_4_-treated electrodes. This means that the mass of adsorbed protein A started plateauing between ca. 80 ng and 130 ng (Table 1—for both examined concentrations), depending on the preconditioning strategy, except for the H_2_SO_4_-treated electrodes (at 20 μg mL^−1^ of protein A) and for positive-plasma-treated electrodes (at 20 μg mL^−1^ of protein A); namely, a positive glow discharge was the only preconditioning strategy where the mass of adsorbed protein A doubled from the lower to the higher applied concentration (i.e., adsorption of 61% and 63% protein A at 10 μg mL^−1^ and 20 μg mL^−1^ applied protein A, respectively). This implies slower adsorption kinetics in the case of positive-plasma-treated electrodes, which is visible also in the electrochemical adsorption kinetic study (Section 3.3). Moreover, the electrochemical study also revealed an unusual pattern of BSA adsorption kinetics at positive-plasma-treated electrodes.

These results are in line with some previous observations [2]. The amount of adsorbed proteins depends on their size, charge, structural stability (“hard” or “soft”), amino acid composition, and steric conformation. BSA is known to have low internal stability and belongs to the so-called “soft” proteins, along with, e.g., immunoglobulins, casein, hemoglobin, and catalase. These proteins adsorb practically on all surfaces regardless of electrostatic interactions and may greatly change their conformation upon adsorption. For BSA, the adsorption has generally been shown to be irreversible [2]. Both the BSA’s structural malleability and tendency towards irreversible adsorption may explain the observed relatively high adsorption degrees that were practically independent of the electrode preconditioning protocol.

Protein A is also known to be structurally flexible, owing to six- to nine-amino-acid long bendable linkers between each of its globular IgG-binding domains [18]. However, our results nevertheless show that its adsorption depended highly on the electrode surface modification. In this regard, protein A interacted with the surface of SPEs more like a “hard” protein, except in the case of positive-plasma-treated electrodes, which follow the concentration dependence similarly as in the case of BSA adsorption.

Notably, an interesting insight can be gained by calculating the mass of proteins adsorbed per electrochemically active area of the working electrode (Table 2). Here, the differences between the two proteins and the behavior described previously become even more apparent. The masses of adsorbed BSA per electrochemically active area at a concentration of 20 μg mL^−1^ are nearly double the masses adsorbed at a concentration of 10 μg mL^−1^ and reach up to 26 ng mm^−2^ for the H_2_SO_4_-treated electrode, whereas the masses of adsorbed protein A per electrochemically active area show a tendency towards plateauing and do not exceed 20 ng mm^−2^. This behavior has implications for the construction of biosensors and for the interpretation of electrochemical readings. From Table 2 and protein molecular weights, we can calculate the number of molecules adsorbed per electrochemically active area, and from protein sizes, we can thus calculate the net surface of adsorbed proteins. For example, BSA (66 kDa) is known to be a prolate ellipsoid with a size of 140 Å (14 nm) at its major axis and 40 Å (4 nm) at its minor axis [19]. If we thus (somewhat simplistically) presume that its side-on orientation takes up 44 nm^2^, we can calculate that in the case of 85% adsorbed BSA (e.g., for a negative-plasma-treated electrode at 20 µg mL^−1^), its surface corresponds to ca. 68 mm^2^, which is about five times larger than the geometric area of the working electrode. This implies that the proteins are not packed horizontally at the electrode surface but form a more complex 3D structure. A high protein surface coverage is also consistent with the electrochemical study carried out in the next section, which shows relatively strong signal attenuations after protein adsorption. This means that to retain suitable sensitivity after the complete sensing architecture is created, one should start with a more sparsely adsorbed base layer. Accordingly, this leaves a higher degree of surface unoccupied and free for non-specific binding of other molecules, especially in real samples, an issue that has been recently addressed in detail by Frutiger et al. [20].

### 3.3. The Effect of Surface Preconditioning on the Adsorption Kinetics of BSA and Protein A—Electrochemical Experiments

**Figure 2:**
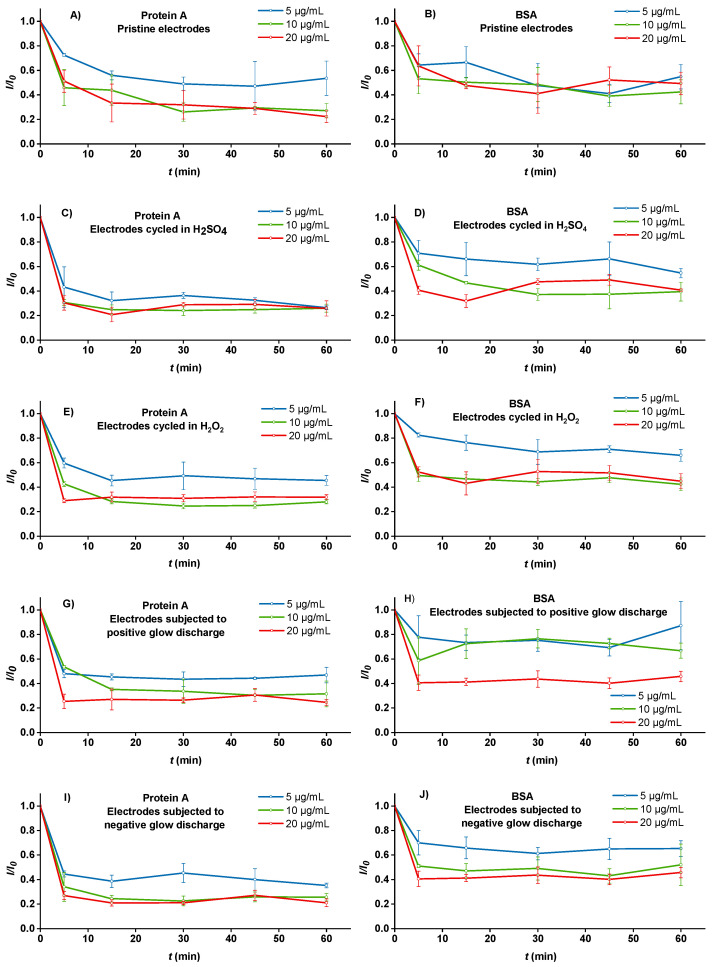
The kinetics of protein A (**A**,**C**,**E**,**G**,**I**) and BSA (**B**,**D**,**F**,**H**,**J**) adsorption at different concentrations (5, 10, and 20 µg mL^−1^) to differently preconditioned SPEs. (**A**,**B**) Pristine electrodes (no preconditioning); (**C**,**D**) H_2_SO_4_-treated electrodes; (**E**,**F**) H_2_O_2_-treated electrodes; (**G**,**H**) positive-plasma-treated electrodes; (**I**,**J**) negative-plasma-treated electrodes. The signal attenuation due to protein adsorption was calculated as *I*/*I*_0_, where *I* is the square-wave voltametric peak current after and *I*_0_ before adsorption at the same electrode recorded in a 1 mM + 1 mM mixture of K_3_[Fe(CN)_6_] and K_4_[Fe(CN)_6_] in 0.1 M KCl. Each time-point is an average of three different electrodes; error bars denote standard deviations.

The electrochemical study (Figure 2) demonstrates that the amount of adsorbed proteins at the SPEs noted in Table 1 does not always straightforwardly translate into the degree of electrochemical signal attenuation, as observed with SWV. Whereas the adsorption kinetics of protein A differs from that of BSA, as also expected from their adsorbed masses, the signal attenuation patterns of electrochemical responses alter much more among different electrode preconditioning strategies than the mass of adsorbed proteins. In addition, the SWV signal attenuation is more pronounced in the case of protein A, particularly in the first 5 min of adsorption, suggesting that the attenuation patterns of SWV signals also involve differences in protein conformation or orientation on the electrode surface and not only their mass. What is more, the adsorption kinetics also cannot be simply ascribed to electrostatic interactions between the proteins and electrode surface, as both proteins are negatively charged at pH 7.5 (at which the experiments were conducted). However, it is clear that all preconditioning strategies improve electrode-to-electrode reproducibility in comparison to pristine electrodes (Figure 1b and Figure 2A, see also error bars), which means preconditioning is an important (if not mandatory) step in the biosensor construction despite the complexity of the factors governing the interactions between proteins and electrode surface.

The first pattern that can be almost universally observed between differently preconditioned electrodes is that the SWV signal attenuation is rapid, reaching the plateau in the first 5 to 15 min of incubation (taking into consideration all three examined concentrations). This is particularly well-pronounced, for example, for the negative-plasma-treated electrodes after the incubation with protein A (Figure 2I). On the other hand, the adsorption of protein A to pristine electrodes was slower and leveled off after approximately 30 min (Figure 2A). Moreover, BSA also showed the fastest, most uniform, and consistent adsorption at the negative-plasma-treated electrodes (Figure 2J).

The next nearly universal observation is the difference in SWV signal attenuation between the electrodes incubated with 5 μg mL^−1^ of either protein on the one hand and the higher two concentrations on the other. The quantification of the adsorbed proteins applied at a concentration of 5 μg mL^−1^ was not possible because the mass of aspired (i.e., non-adsorbed) proteins would fall below the limit of detection of the FluoroRed 600 assay. However, based on Table 1, we can easily speculate that the majority of proteins at this concentration became adsorbed. The signal attenuation patterns at the concentration of 5 μg mL^−1^ generally show fast and stable adsorption, except for pristine electrodes, where the adsorption of protein A followed a parabolic curve (Figure 2A) and the adsorption of BSA was uneven (Figure 2B), again implying the importance of surface preconditioning. The degree of final signal attenuation was generally lower than at the higher two concentrations, ranging from as low as 25% in the case of BSA adsorbed to the positive-plasma-treated electrodes (Figure 2H) to as high as 75% in the case of protein A adsorbed to the H_2_SO_4_-treated electrodes after 60 min incubation (Figure 2C). Evidently, the adsorption of protein A at the concentration of 5 μg mL^−1^ was relatively less affected by different preconditioning approaches than in the case of BSA, which exhibited the highest and the most stable adsorption in the case of negative-plasma-treated electrodes.

The adsorption kinetics of both proteins at concentrations of 10 and 20 μg mL^−1^ generally resulted in a similar degree of SWV signal attenuation (60–80%), with the exception of BSA adsorbed to the positive-plasma-treated electrodes, where the highest concentration caused almost twice as intense (and stable with time) attenuation as the lower two BSA concentrations exhibiting also less stable signals (Figure 2H). Although the observations are somewhat more consistent with the mass of adsorbed proteins in the case of protein A (Table 1), they are more difficult to explain in the case of BSA, where the masses of adsorbed BSA consistently increased with their higher concentration in the modification solution. These phenomena imply a stronger effect of protein A upon the electrochemical response along with the importance of surface preconditioning.

The adsorption kinetics of both proteins at a concentration of 20 μg mL^−1^ in some instances (particularly in Figure 2C–F) show an interesting course that is described in the literature as “overshooting kinetics”. Overshooting kinetics is a consequence of either the change of the protein’s conformation in contact with the surface or its rearrangement from end-on to side-on position with time [21]. Regardless of its origin, the occurrence of overshooting kinetics demonstrates that the protein layer adsorbed to the tested SPEs was, in some cases, unstable for at least 45 min. Therefore, at least 60 min are needed for the basal protein layer to establish itself before another protein layer should be added. Protein rearrangements on the surface are more pronounced at their higher solution concentrations [2]; thus, where non-specific binding is not a concern [20], lower protein concentrations may suffice for the construction of the basal layer as they may help both limit the occurrence of overshooting kinetics, reduce material consumption, and provide a higher electroanalytical signal of a specific redox probe used.

### 3.4. The Effect of Surface Preconditioning on the Protein–Surface Interaction

**Figure 3:**
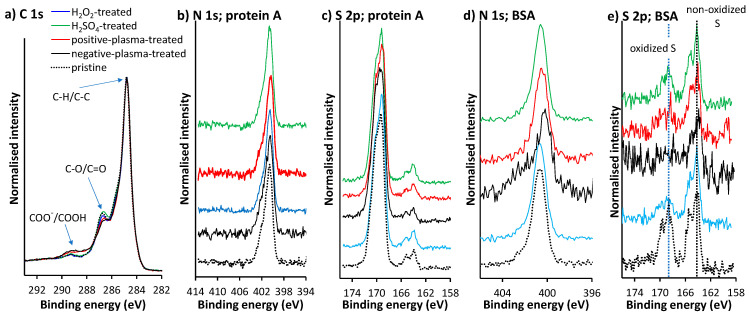
(**a**) High-resolution C 1s spectra for differently activated electrodes before protein adsorption. High-resolution N 1 s XPS spectra after (**b**) protein A and (**d**) BSA adsorption, and S 2p XPS spectra measured after (**c**) protein A and (**e**) BSA adsorption.

Table 3 shows the atomic surface concentrations of the differently preconditioned electrodes; the analyses were performed on the surfaces of the SPE working electrodes. The surfaces consisted of C-, O-, Si-, and Cl-containing species. C and O originate mainly from the SPE working electrode. The contribution to the C and O signals, and consequently the increased surface concentrations of these elements, may also come from the adventitious carbonaceous species (non-oxidized and oxidized) that adsorbed on the surface after sample preparation and during sample transfer to the spectrometer. Cl- and Si-containing species originated from the SPE binder. The most significant difference between the activation methods used is in the O concentration, which was higher for both plasma-treated surfaces (Table 3). When the plasma sources were used, the surface modification resulted mainly in the formation of a higher surface concentration of carboxyl groups and a lower surface concentration of C=O/C-O groups, as shown in Figure 3a.

The XPS technique was also used to study the adsorption of protein A and BSA on the differently activated SPE electrodes. A drop of the PBS solution containing protein A and BSA at a concentration of 10 μg mL^−1^ was pipetted onto each of the five SPEs electrodes. The drop was left on the electrodes for 1 h. After that, the electrodes were rinsed with ultrapure water and wiped with sensitive cleaning wipes (Kimtech Science, Kimberly Clark Worldwide, Irving, TX, USA) without touching the active area of the working electrode. Then the electrodes were placed in the XPS spectrometer. Figure 3b–e show that N 1s and S 2p signals were present for all five electrodes, confirming protein A and BSA adsorption on the working electrodes. The N 1s and S 2p spectra for five electrodes with adsorbed protein A had a similar shape and position, indicating a similar N and S atoms environment when adsorbed on the surface. On the other hand, when adsorbed on the negative-plasma-treated surface, the environment of N and S atoms for BSA is different from the other four electrodes. The N 1s peak is shifted to a more negative *E*_B_ for the negative-plasma-treated electrode compared to the other four electrodes. In addition, the S 2p spectra in Figure 3e contain features corresponding to oxidized S atoms (at more positive *E*_B_) and non-oxidized S atoms (at more negative *E*_B_), both indicated by a dotted vertical line. The feature corresponding to oxidized S atoms was not developed in the negative-plasma-treated electrode, whereas the feature for non-oxidized S was present. For the other four electrodes, the feature for oxidized S appeared with the most intense peak for the pristine electrode, followed by the H_2_SO_4_-treated, the H_2_O_2_-treated, and the positive-plasma-treated electrode. The latter indicates that BSA adsorption at the negative-plasma-treated electrode occurs via different binding centers compared to BSA adsorption for the other four electrodes.

## 4. Conclusions

Several factors are important when choosing an appropriate combination of protein concentration and electrode surface preconditioning strategy for the purpose of biosensor construction. In combination with a screen-printed supporting carbon electrode and protein A and BSA, as two typical model proteins, we studied the effect of different preconditioning strategies, such as cyclic voltametric pretreatment in H_2_SO_4_ and H_2_O_2_ and plasma pretreatment with a positive and negative glow discharge. The XPS spectra of the preconditioned SPE surfaces (before protein adsorption) revealed that the most significant difference between the activation methods used was in the O concentration, which was higher for both plasma-treated surfaces and was attributed to the formation of carboxyl groups. In addition, after the protein adsorption, the feature corresponding to oxidized S atoms was not developed at the negative-plasma-treated electrode in combination with BSA adsorption, indicating the BSA adsorption via different binding centers compared to BSA adsorption at the other examined electrodes. The study showed that after preconditioning, for both protein A and BSA, the concentrations of 5 and 10 μg mL^−1^ generally yield a stably adsorbed protein layer after 60 min of incubation time, except for BSA at the positive-plasma-treated electrode. On the other hand, 20 μg mL^−1^ may for both proteins result in a layer that is prone to structural rearrangements at the electrode surface (as evident from the overshooting kinetics); however, the choice of concentration depends on the desired degree of surface coverage and/or attenuation of the electrochemical signal. Electrode preconditioning typically accelerated the adsorption kinetics and, most importantly, improved electrode-to-electrode reproducibility; yet, its choice depends on the available equipment and the protein in question, as protein A and BSA interact differently with differently pretreated electrodes. In addition, protein A exhibited a stronger effect on the electrochemical signal attenuation than BSA when applying the same protein concentrations. For protein A, all tested preconditioning strategies were found to be useful, particularly the negative glow discharge when comparing all three examined concentrations (5, 10, and 20 μg mL^−1^) versus electrochemical stability. For BSA, however, the only generally beneficial preconditioning strategy was the negative glow discharge, whereas others showed different effects in relation to the protein concentration, especially at the lowest examined BSA concentration of 5 μg mL^−1^ with as low as 25% signal attenuation in the case of BSA adsorbed to the positive-plasma-treated electrodes. Moreover, when taking into account the relatively sluggish adsorption kinetics of protein A at the positive-plasma-treated electrode, we can conclude that negative-plasma-treated electrodes exhibit the most favorable electrochemical stability, particularly when simultaneously considering both proteins at their different concentrations.

## Figures and Tables

**Table 1:** The amount of proteins adsorbed on the working electrode of SPEs in relation to the surface preconditioning strategy and protein concentration. The values reported are averages of two replicates.

Electrode Preconditioning	Percentage of the Adsorbed Protein (%)			
	BSA		Protein A	
	10 μg mL^−1^ (100 ng in 10 μL)	20 μg mL^−1^ (200 ng in 10 μL)	10 μg mL^−1^ (100 ng in 10 μL)	20 μg mL^−1^ (200 ng in 10 μL)
None (pristine electrodes)	85	84	84	60
H_2_O_2_-treated	92	79	≥98 *	66
H_2_SO_4_-treated	81	86	85	33
Positive-plasma-treated	87	86	61	63
Negative-plasma-treated	84	85	≥82 *	47

* The mass of proteins aspirated from one of the electrodes was below the detection limit of the method.

**Table 2:** The mass of proteins adsorbed per electrochemically active area of the working electrode of SPEs in relation to the surface preconditioning strategy and protein concentration. The values are recalculated from Table 1, and the electrochemically active areas are reported in Section 3.1.

Electrode Preconditioning	The Adsorbed Protein Mass per Electrochemically Active Area (ng mm^−2^)			
	BSA		Protein A	
	10 μg mL^−1^ (100 ng in 10 μL)	20 μg mL^−1^ (200 ng in 10 μL)	10 μg mL^−1^ (100 ng in 10 μL)	20 μg mL^−1^ (200 ng in 10 μL)
None (pristine electrodes)	10	20	10	14
H_2_O_2_-treated	14	24	≥15 *	20
H_2_SO_4_-treated	12	26	13	10
Positive-plasma-treated	12	23	8	17
Negative-plasma-treated	12	24	≥12 *	13

* The mass of proteins aspirated from one of the electrodes was below the detection limit of the method.

**Table 3:** Measured atomic surface concentrations of differently treated working electrodes.

Electrode Preconditioning	C [at. %]	O [at. %]	Si [at. %]	Cl [at. %]
None (pristine electrode)	86.1	5.6	1.5	6.8
H_2_O_2_-treated	86.9	5.3	0.9	6.8
H_2_SO_4_-treated	86.9	5.4	1.2	6.6
Positive-plasma-treated	83.0	10.6	1.4	5.0
Negative-plasma-treated	80.7	13.3	1.9	4.0

## Supplementary Materials

The following supporting information can be downloaded at: https://www.mdpi.com/article/10.3390/s22114186/s1, Figure S1: Cyclic voltammograms at pristine (A), H_2_SO_4_-treated (B), H_2_O_2_-treated (C), positive-plasma-treated (D), and negative-plasma-treated (E) electrodes obtained in a 1 mM + 1 mM mixture of K_3_[Fe(CN)_6_] and K_4_[Fe(CN)_6_] in 0.1 M KCl together with the corresponding graphs of current (I) vs. square root of the scan rate (*υ*^½^); Figure S2: The stability study of the electrochemical signal at a SPE during 2 × 25 consecutive SWV scans (potential range of −0.4 V to 0.8 V, step potential = 0.01 V, amplitude = 0.05 V, frequency = 20 Hz) in a 1 mM + 1 mM mixture of K_3_[Fe(CN)_6_] and K_4_[Fe(CN)_6_] in 0.1 M KCl, at ambient conditions. The first 25 uninterrupted SWV scans (A), and the next 25 SWV scans during which the electrode was removed from the solution after every 5 scans, washed with deionized water, and dried in nitrogen (B). Red arrows show the washing and drying events; Figure S3: The stability study of the electrochemical signal at SPEs during 2 × 25 consecutive SWV scans (conditions are as in Figure S2). The electrodes electrochemically cycled in either 10 mM H_2_SO_4_ (A,B) or 1 mM H_2_SO_4_ (C,D) were first subjected to 25 SWV scans during which the electrodes were removed from the solution after every 5 scans, washed with deionized water, and dried in nitrogen (A,C). Red arrows show the washing and drying events. This was followed by 25 uninterrupted SWV scans (B,D); Figure S4: The stability study of the electrochemical signal at a SPE during 2 × 25 consecutive SWV scans (conditions are as in Figure S2). The electrode electrochemically cycled in 10 mM H_2_O_2_ was first subjected to 25 SWV scans during which the electrode was removed from the solution after every 5 scans, washed with deionized water, and dried in nitrogen (A). Red arrows show the washing and drying events. This was followed by 25 uninterrupted SWV scans (B); Figure S5: The stability study of the electrochemical signal at SPEs during 2 × 25 consecutive SWV scans (conditions are as in Figure S2). The electrodes preconditioned with either negative (A) or positive (C) glow discharge were first subjected to 25 SWV scans during which the electrodes were removed from the solution after every 5 scans, washed with deionized water, and dried in nitrogen. Red arrows show the washing and drying events. This was followed by 25 uninterrupted SWV scans (B,D).
