# Supplementary material for: The Effect of Preconditioning Strategies on the Adsorption of Model Proteins onto Screen-Printed Carbon Electrodes
Source: Sensors (Basel). 2022 May 31;22(11):4186. doi: 10.3390/s22114186 (PMC9185278; doi:10.3390/s22114186)
Supplement: Supplementary file 1 [file sensors-22-04186-s001.zip › sensors-1733344-supplementary.pdf]

Supplementary Materials

The Effect of Preconditioning Strategies on the Adsorption of Model Proteins onto Screen-Printed Carbon Electrodes

Tea Romih [1], Ivan Konjević [2], Lea Žibret [1], Ika Fazarinc [1], Ajda Beltram [1], David Majer [2], Matjaž Finšgar [2] and Samo B. Hočevar [1,*]

[1] Department of Analytical Chemistry, National Institute of Chemistry, Hajdrihova 19, SI-1000 Ljubljana, Slovenia; tea.romih@ctk.uni-lj.si (T.R.); lea.zibret@zag.si (L.Ž.); if2912@student.uni-lj.si (I.F.); ab9644@student.uni-lj.si (A.B.)

[2] Faculty of Chemistry and Chemical Technology, University of Maribor, Smetanova 17, SI-2000 Maribor, Slovenia; ivan.konjevic@student.um.si (I.K.); david.majer@um.si (D.M.); matjaz.finsgar@um.si (M.F.)

* Correspondence: samo.hocevar@ki.si; Tel.: +386-1-4760-230

S1. Assessment of Electrochemically Active Surface Area

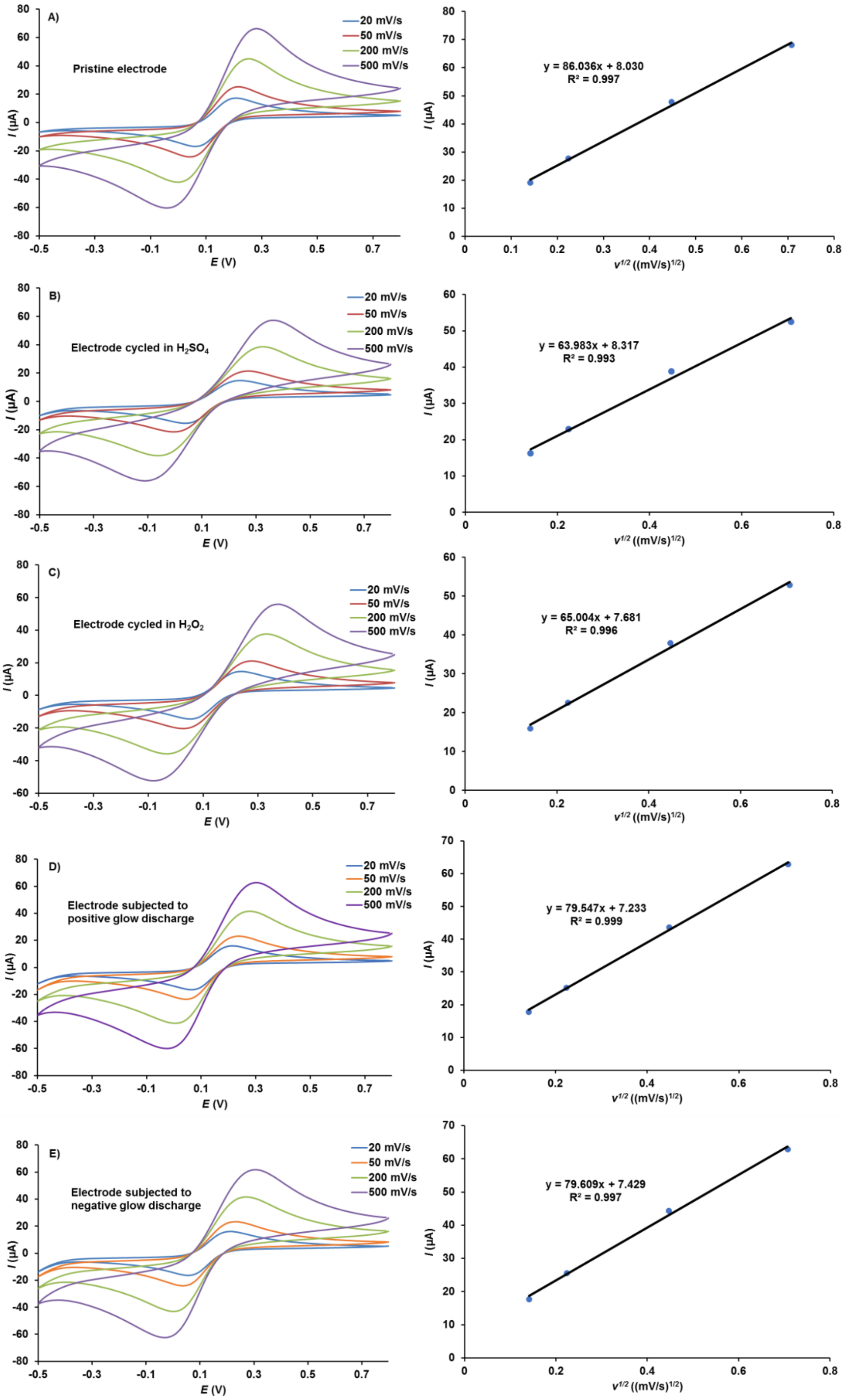

Figure S1. Cyclic voltammograms at pristine (A), H_2SO_4-treated (B), H_2O_2-treated (C), positive-plasma-treated (D), and negative-plasma-treated (E) electrodes obtained in a 1 mM + 1 mM mixture of $K_3[Fe(CN)_6]$ and $K_4[Fe(CN)_6]$ in 0.1 M KCl together with the corresponding graphs of current (I) vs. square root of the scan rate ($\upsilon^{1/2}$).

S2. The Stability Study of a Square-Wave Voltametric Signal of Pristine Screen-Printed Carbon Electrodes Through Consecutive Measurements

A pristine Metrohm DropSens DRP-C110 screen-printed electrode (SPE) was subjected to 2×25 consecutive square-wave voltametric (SWV) scans in a potential range of −0.4 V to +0.8 V, in a 1 mM + 1 mM mixture of $K_3[Fe(CN)_6]$ and $K_4[Fe(CN)_6]$ in 0.1 M KCl. The measurements were performed in a 10 mL glass electrochemical cell with the aid of a cable connector for SPEs (DRP-CAC 71606, Metrohm DropSens). The electrode was first subjected to 25 consecutive SWV scans uninterruptedly (Figure S2A), after which it was removed from the solution after every 5 scans, washed with deionized water, and dried in nitrogen before resuming the measurements (Figure S2B).

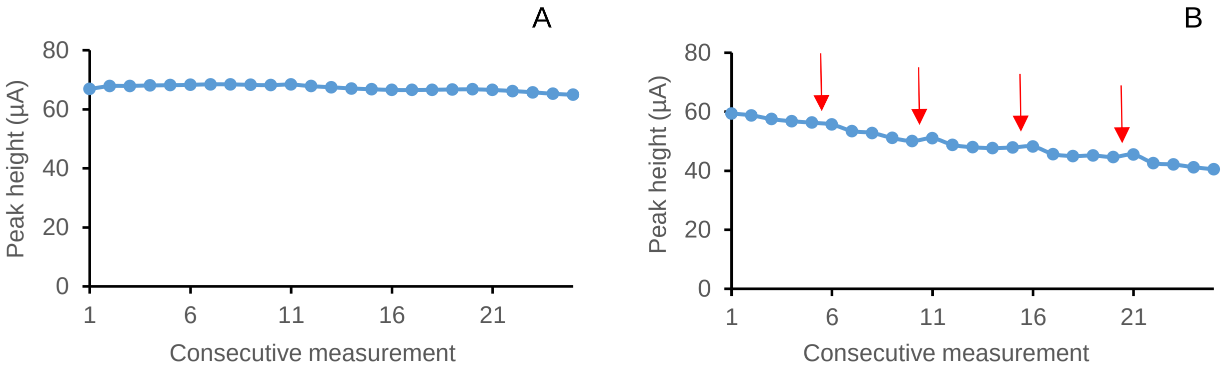

Figure S2. The stability study of the electrochemical signal at a SPE during 2×25 consecutive SWV scans (potential range of −0.4 V to 0.8 V, step potential = 0.01 V, amplitude = 0.05 V, frequency = 20 Hz) in a 1 mM + 1 mM mixture of $K_3[Fe(CN)_6]$ and $K_4[Fe(CN)_6]$ in 0.1 M KCl, at ambient conditions. The first 25 uninterrupted SWV scans (A), and the next 25 SWV scans during which the electrode was removed from the solution after every 5 scans, washed with deionized water, and dried in nitrogen (B). Red arrows show the washing and drying events.

The results shown in Figure S2A clearly demonstrate that the electrochemical signal was predominantly stable when the electrode was used uninterruptedly, whereas it gradually decreased for approximately 20 µA (ca. 33%) from the 26th to the 50th measurement when the SPE was removed from the solution, dried, and reused (Figure S2B). Since this is likely to happen if SPEs are used for the construction of biosensors, the study of preconditioning via potential cycling in H_2SO_4 and H_2O_2, and plasma preconditioning (with positive and negative glow discharge) was carried out, aimed at stabilizing the electrochemical signal during electrode reuse.

S3. The Stability Study of the Electrochemical Signal of the SPEs Preconditioned In H_2SO_4

The parameters for potential cycling in H_2SO_4 were chosen as stringent enough to achieve the desired effect but milder than for, e.g., glassy carbon electrodes. In our practical experience, the most problematic part of SPEs is the silver quasi-reference electrode, which may react with concentrated acid; to avoid this, we used diluted H_2SO_4. The potential cycling parameters were as follows: 3 voltammetric cycles in a potential window of –1.5 V to 1.5 V, step potential of 0.01 V, and a scan rate of 0.1 V/s. Then the electrodes were tested similarly as described in Section S2, but the rinsing and drying set was done first, followed by uninterrupted measurements, to directly demonstrate the effect of preconditioning. The measurements after preconditioning the SPEs in 10 mM and 1 mM H_2SO_4 are shown in Figure S3.

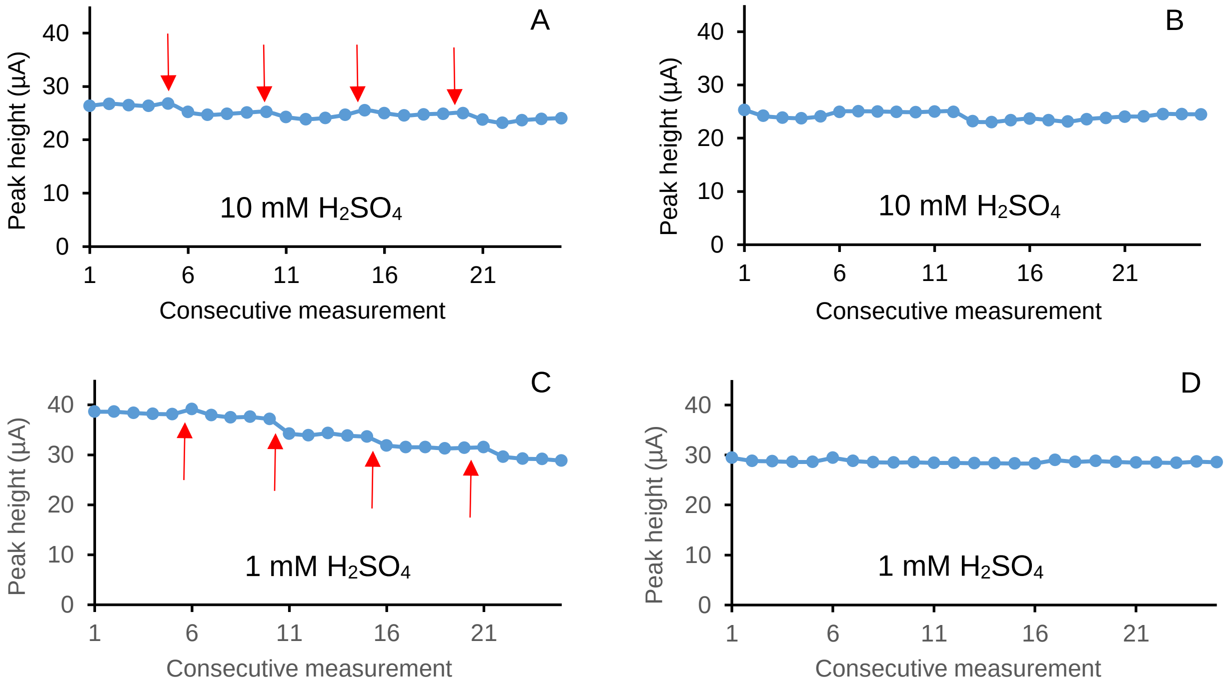

Figure S3. The stability study of the electrochemical signal at SPEs during 2×25 consecutive SWV scans (conditions are as in Figure S2). The electrodes electrochemically cycled in either 10 mM H_2SO_4 (A, B) or 1 mM H_2SO_4 (C, D) were first subjected to 25 SWV scans during which the electrodes were removed from the solution after every 5 scans, washed with deionized water, and dried in nitrogen (A, C). Red arrows show the washing and drying events. This was followed by 25 uninterrupted SWV scans (B, D).

The results show that during interrupted measurements, the electrochemical signal fluctuated in a similar manner as at pristine electrodes but decreased to a lesser degree (Figures S3A and S3C vs Figure S2B). Although the signal of the electrode that was electrochemically cycled in 1 mM H_2SO_4 (Figure S3C) attenuated more during repeated washing and drying cycles in comparison to the electrode that was cycled in 10 mM H_2SO_4, (Figure S3A), the peak height of the latter was somewhat lower, and the signal during uninterrupted measurements was less stable (Figure S3B). We therefore adopted the electrochemical cycling in a more diluted acid for our further experiments involving protein adsorption.

S4. The Stability Study of the Electrochemical Signal of the SPE Preconditioned In H_2O_2

The electrodes electrochemically cycled in H_2O_2 were tested in the same way as those cycled in H_2SO_4. In this case, the electrochemical signal fluctuated the most of all preconditioning strategies (Figure S4). The fluctuation seemed to be random, not correlating with the washing/drying events (Figure S4A), and remained relatively high during the uninterrupted measurements (Figure S4B).

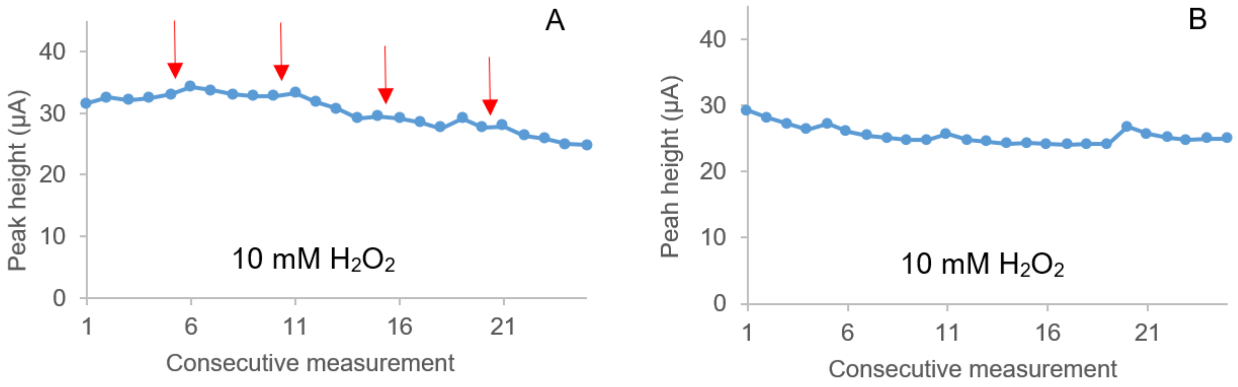

Figure S4. The stability study of the electrochemical signal at a SPE during 2×25 consecutive SWV scans (conditions are as in Figure S2). The electrode electrochemically cycled in 10 mM H_2O_2 was first subjected to 25 SWV scans during which the electrode was removed from the solution after every 5 scans, washed with deionized water, and dried in nitrogen (A). Red arrows show the washing and drying events. This was followed by 25 uninterrupted SWV scans (B).

S5. The Stability Study of the Electrochemical Signal of the SPEs Subjected to Positive or Negative Glow Discharge

We performed the same testing of the electrochemical signal stability also for the SPEs that were subjected to positive or negative glow discharge (Figure S5). The electrochemical response of the electrodes that were subjected to the positive glow discharge was the most stable of all examined preconditioning strategies during the rinsing and drying experiment (Figure S5C), whereas the signal at the electrodes subjected to the negative glow discharge was slightly less stable (Figure S5A). However, the electrochemical signals during uninterrupted measurements remained satisfactorily stable in both cases (Figures S5B and S5D). We can conclude that the highest overall electrochemical stability of the SPEs can be achieved using plasma preconditioning.

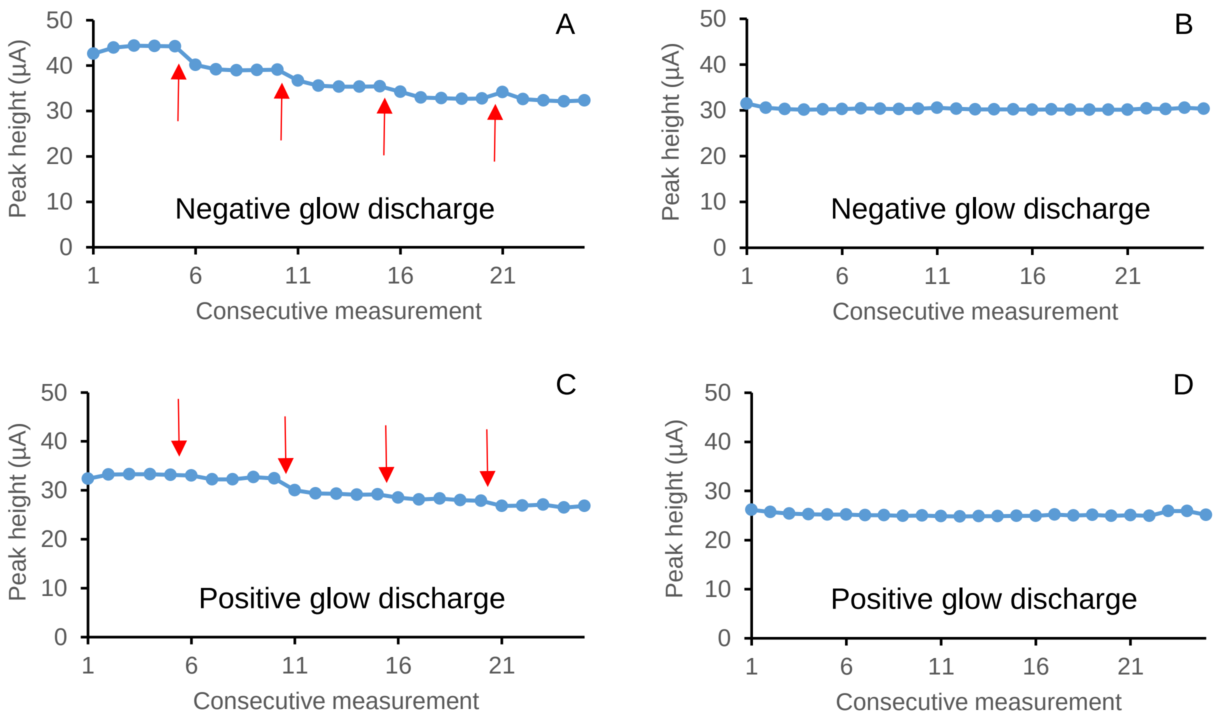

Figure S5. The stability study of the electrochemical signal at SPEs during 2×25 consecutive SWV scans (conditions are as in Figure S2). The electrodes preconditioned with either negative (A) or positive (C) glow discharge were first subjected to 25 SWV scans during which the electrodes were removed from the solution after every 5 scans, washed with deionized water, and dried in nitrogen. Red arrows show the washing and drying events. This was followed by 25 uninterrupted SWV scans (B and D).
